# The Contribution of Surface Tension-Dependent Alveolar Septal Stress Concentrations to Ventilation-Induced Lung Injury in the Acute Respiratory Distress Syndrome

**DOI:** 10.3389/fphys.2020.00388

**Published:** 2020-06-26

**Authors:** Carrie E. Perlman

**Affiliations:** Department of Biomedical Engineering, Stevens Institute of Technology, Hoboken, NJ, United States

**Keywords:** acute respiratory distress syndrome (ARDS), mechanical ventilation, ventilation-induced lung injury (VILI), surface tension, stress concentrations, recruitment maneuvers

## Abstract

In the acute respiratory distress syndrome (ARDS), surface tension, *T*, is likely elevated. And mechanical ventilation of ARDS patients causes ventilation-induced lung injury (VILI), which is believed to be proportional to *T*. However, the mechanisms through which elevated *T* may contribute to VILI have been under-studied. This conceptual analysis considers experimental and theoretical evidence for static and dynamic mechanical mechanisms, at the alveolar scale, through which elevated *T* exacerbates VILI; potential causes of elevated *T* in ARDS; and *T*-dependent means of reducing VILI. In the last section, possible means of reducing *T* and improving the efficacy of recruitment maneuvers during mechanical ventilation of ARDS patients are discussed.

## Introduction

In healthy lungs, the alveoli are lined by a thin, aqueous liquid lining layer with associated surface tension, *T* (Gil et al., 1979). Surface tension acts in parallel with lung tissue elasticity to tend to collapse the lungs. The impact of *T* is lessened by alveolar epithelial type II cell secretion of pulmonary surfactant, a complex mixture of phospholipids, neutral lipids, and hydrophobic and hydrophilic proteins (Haagsman and van Golde, 1991). Surfactant reduces *T* at the alveolar interface, yet *T* varies cyclically as lung inflation decreases interfacial surfactant concentration and increases *T* (Kharge et al., 2014). Surfactant is first produced in the third trimester of gestation. In neonatal respiratory distress syndrome (NRDS), babies born severely prematurely have reduced levels of lung surfactant, thus high surface tension in their lungs, and are unable to breathe unassisted (Clements, 1997). This situation demonstrates the important contribution of *T* to lung mechanics.

In adults, a variety of pulmonary or systemic insults—e.g., pneumonia caused by a corona-virus, gastric aspiration, sepsis, or acute pancreatitis—can lead to acute respiratory distress syndrome (ARDS) (Ware, 2006; Wu et al., 2020). In ARDS, regardless of initial insult, pulmonary inflammation is present and causes an increase in alveolar–capillary barrier permeability. Barrier permeability leads first to interstitial and then to alveolar edema. The edema liquid, which contains plasma proteins, impairs gas exchange. Additionally, in ARDS, alveolar *T* is believed to be elevated (Günther et al., 1996; Holm et al., 1999).

Both NRDS and ARDS patients are treated by mechanical ventilation, which supports gas exchange but also causes an exacerbation of underlying lung injury—known as ventilation-induced lung injury (VILI)—and can prevent patient recovery (Jobe et al., 1983; Brower et al., 2000; Cereda et al., 2017). In NRDS, surfactant therapy, i.e., tracheal instillation of exogenous surfactant, is a successful intervention that supplements the low surfactant level of immature lungs and reduces or prevents VILI (Clements, 1997). Stemming from the success in NRDS, it is widely believed that surfactant therapy should likewise reduce VILI in ARDS. However, surfactant therapy has failed in clinical trials to reduce ARDS mortality (Brower and Fessler, 2011).

The failure of surfactant therapy for ARDS raises doubts about the connection between *T* and VILI, and the mechanical mechanism through which *T* may influence VILI is not fully understood. While there have been excellent studies of *T* effects on the mechanics of injured whole lungs (e.g., Bachofen et al., 1987; Gommers et al., 1993; Yamashita et al., 2008), *T* effects on alveolar septal micromechanics in the presence of injury or edema have been under-studied. Following below is a conceptual analysis of the influence of *T* on septal mechanics in injured regions, causes of elevated *T* in ARDS, and possible means of reducing VILI by reducing *T*-dependent septal stress concentrations in injured regions.

## Mechanical Mechanisms of Ventilation-Induced Lung Injury and the Effects of Surface Tension

If mechanical properties were homogenous throughout the parenchyma of injured lungs, then elevated *T* would uniformly restrain the lungs and be protective. As mechanical properties throughout injured lungs are heterogeneous, however, there are stress concentrations present that are proportional to *T* and can be exacerbated by elevated *T*.

### Injury Heterogeneity

Injury throughout ARDS lungs is heterogeneous in type and in spatial distribution. Types of parenchymal injury include, but are not limited to: (i) edematous alveoli flooded with proteinaceous blood plasma; (ii) hemorrhagic alveoli flooded with whole blood, including lysed red blood cells (RBCs); and (iii) collapsed alveoli (Staub et al., 1967; Henzler et al., 2011; Santos et al., 2012; de Prost et al., 2014). By microscopy of the injured lung surface, flooding and hemorrhage have been observed. By histology, all three forms of injury have been observed (Wu et al., 2017).

Spatial heterogeneity of injury is evident at scales from that of the alveolus to that of the whole lung. At the alveolar scale, histologic analysis shows that edema, hemorrhage, and collapse are all present in a heterogeneous fashion, interspersed with aerated alveoli (Staub et al., 1967; Henzler et al., 2011; Santos et al., 2012; de Prost et al., 2014). At an intermediate scale, the surfaces of lungs excised after 5 min of ventilation with a high peak inspiratory pressure (PIP) of 45 cm H_2_O exhibit focal regions of injury surrounded by apparently healthy parenchyma (Dreyfuss and Saumon, 1998). And at the scale of the whole lung, computed tomography (CT) shows edema/collapse to be prevalent in the dependent lung (Gattinoni et al., 2001).

### Static, *T*-Dependent Stress Concentrations Due to Injury Heterogeneity

**FIGURE 1:**
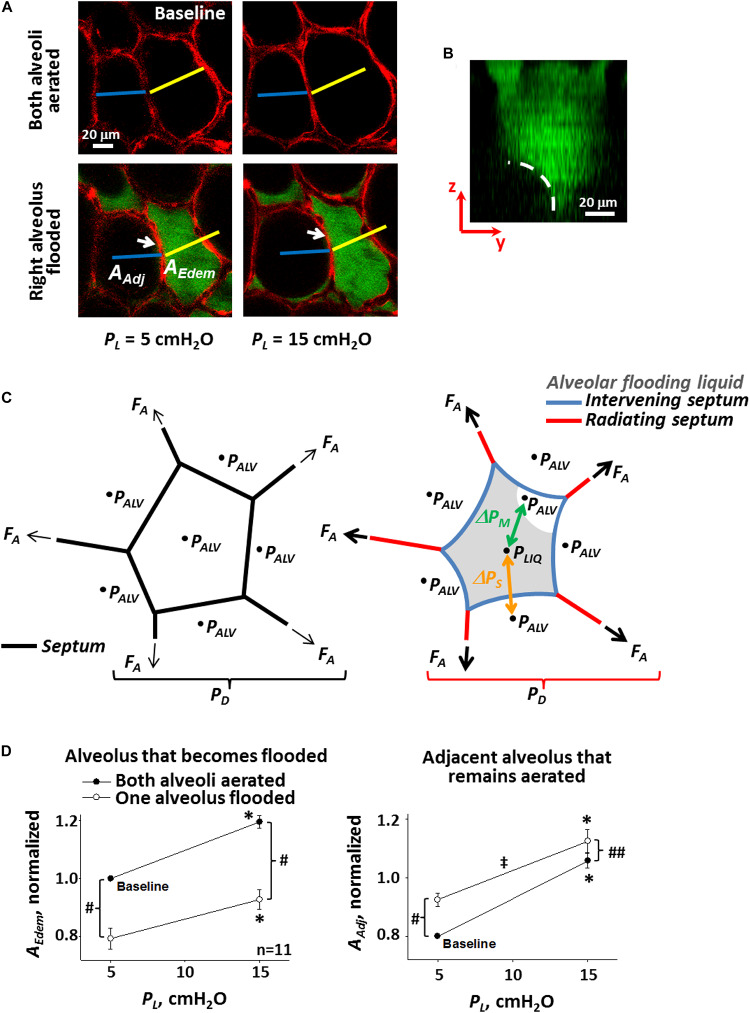
Single alveolar edema model. **(A)** Inflation and flooding effects on alveolar geometry. Optical sections of two surface alveoli in isolated, perfused rat lungs. Epithelium labeled with calcein red-orange. Transpulmonary pressure, *P*_*L*_, as indicated. In bottom images, right alveolus flooded with fluorescein-labeled 5% albumin solution. Air appears black. Sections are 2-μm thick and at 20-μm sub-pleural depth. Flooding right alveolus causes bowing of central “intervening” septum that separates aerated and flooded alveoli (arrows). Scale bars from baseline image are superimposed on all other images. Right alveolus that becomes flooded and left alveolus that remains aerated have areas *A*_*Edem*_ and *A*_*Adj*_, respectively. **(B)** Meniscus in flooded alveolus. Image is *y–z* section, constructed from confocal *z*-stack, of an alveolus flooded with fluorescein-labeled 5% albumin solution. Pleural surface is at top of image. Epithelium is unlabeled and, along with air, appears black. Dashed line shows meniscus at mouth of flooded alveolus. **(C)** Schematics of alveolus before (left) and after (right) liquid flooding. Intervening septa separate aerated from flooded alveoli. Radiating septa are directed outward from flooded alveolus and have aerated alveoli to each side. *P*_*ALV*_ is alveolar air pressure. *P*_*LIQ*_ is flooding liquid pressure. Air–liquid interface in flooded alveolus forms a meniscus. Δ*P*_*M*_ = *P*_*ALV*_ – *P*_*LIQ*_ is pressure drop across meniscus. Δ*P*_*S*_ = *P*_*ALV*_ – *P*_*LIQ*_ is pressure drop across intervening septa. Axial distending force *F*_*A*_ acts at ends of sectioned septa and is greater in radiating septa around flooded alveolus on the right than in normally-stressed septa around aerated alveolus on the left. Ratio of summed *F*_*A*_ over alveolar surface is an effective distending pressure *P*_*D*_ applied to central alveolus. **(D)** Alveolar compliance. Alveolar areas plotted vs. *P*_*L*_ for pairs of adjacent alveoli, one of which becomes flooded as in **(A)**. Slope of lines is a two-dimensional analog of compliance. Statistics: ^∗^Area greater at *P*_*L*_ of 15 than 5 cm H_2_O (*p* < 0.01), for state before or after one alveolus is flooded. ^#^Area different after than before one alveolus is flooded, at constant *P*_*L*_ (*p* < 0.01). ^##^Same as “#” but *p* < 0.02. ^‡^Slope less after than before one alveolus is flooded (*p* < 0.01). **(A,B,D)** Modified, with permission, from Perlman et al. (2011).

At the alveolar scale, with the lungs held at constant volume, alveoli that are flooded are diminished in size (Figure 1A) (Staub et al., 1967; Perlman et al., 2011). The diminishment is attributable to the interface of the flooding liquid forming a meniscus at the alveolar mouth (Figure 1B) (Bachofen et al., 1993; Perlman et al., 2011; Rühl et al., 2019) and the fact that there is a pressure drop, Δ*P*_*M*_, across the meniscus. Consider an alveolus that is initially aerated and surrounded by aerated neighbors; air pressure in all alveoli is *P*_*ALV*_ (Figure 1C, left). As air pressure is the same across all septa, septa are planar. When the central alveolus becomes flooded, air pressure above the meniscus and in surrounding alveoli remains *P*_*ALV*_. However, according to the Laplace relation, Δ*P*_*M*_ = *P*_*ALV*_ − *P*_*LIQ*_ = 2*T*/*R*, where *P*_*LIQ*_ is liquid pressure below the meniscus, and *R* is meniscus radius of the curvature, such that *P*_*LIQ*_ < *P*_*ALV*_ (Figure 1C, right). As a result of the lower pressure within than around the flooded alveolus, the flooded alveolus decreases in size. In particular, the decrease is often due to bowing into the flooded alveolus of “intervening” septa (Figure 1A, arrows; Figure 1C, blue septa) (Staub et al., 1967; Perlman et al., 2011) separating the flooded alveolus from surrounding aerated alveoli. Intervening septal bowing is caused by pressure difference Δ*P*_*S*_, equal to higher *P*_*ALV*_ in the aerated alveolus to one side minus lower *P*_*LIQ*_ in the flooded alveolus to the other side. The bowing over-extends intervening septa beyond their length when they are planar in normal, aerated lungs, making intervening septa sites of stress concentration. Further, Δ*P*_*S*_ = *P*_*ALV*_ − *P*_*LIQ*_ = Δ*P*_*M*_ ∼ *T*. Thus, intervening septa are over-extended to a degree ∼*T*.

Alveolar derecruitment and collapse have also been attributed to elevated *T* (Albert et al., 2019; Kiefmann et al., 2019; Rühl et al., 2019). And diminishment of an individual alveolus tends, by pulling inward, to over-extend “radiating” septa that splay outward through the surrounding aerated parenchyma (Figure 1C, red septa). Thus, as supported by modeling (Mead et al., 1970; Albert et al., 2019), radiating septa are likely another site of *T-*dependent stress concentration. At the same time as a diminished alveolus pulls inward on radiating septa, each radiating septum transmits a higher-than-normal axial distending force, *F*_*A*_, to the diminished alveolus. The ratio of the summed *F*_*A*_ around an alveolus to the surface area (or in a two-dimensional analog, perimeter length) of that alveolus applies an effective outward distending pressure, *P*_*D*_, to the alveolus, and a diminished alveolus is subject to a greater *P*_*D*_ than a normal-sized alveolus (Mead et al., 1970; Albert et al., 2019).

**FIGURE 2:**
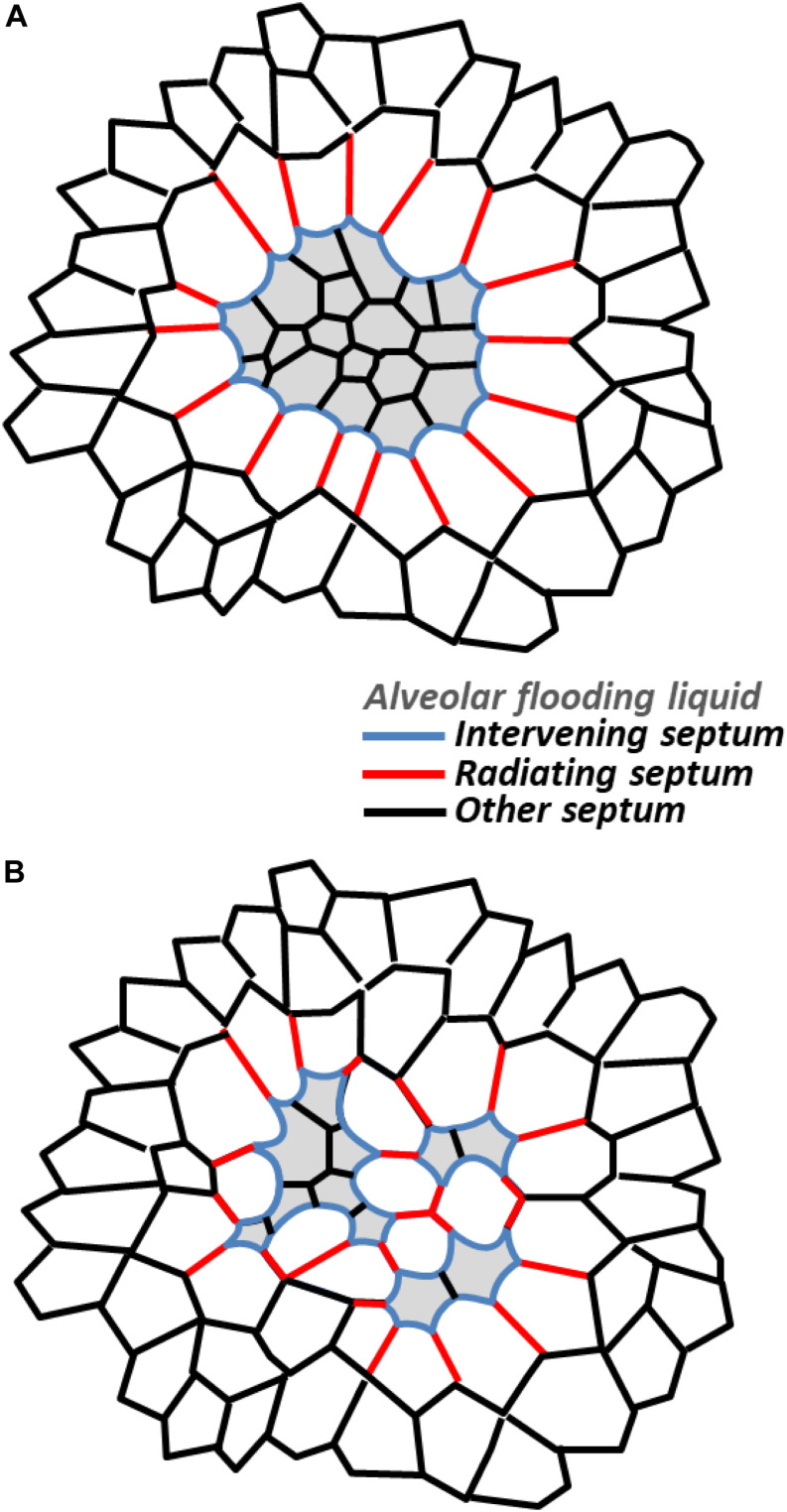
Stress concentrations in injured regions. Schematic representations showing stressed intervening and radiating septa in **(A)** homogeneously and **(B)** heterogeneously flooded regions. While only single layers of radiating septa are shown, computational modeling indicates radiating septa, especially those around a larger injured area, penetrate further into surrounding, aerated parenchyma (see text).

At the intermediate scale, a region of homogeneously flooded or collapsed alveoli should have the effect of a giant, individual flooded or collapsed alveolus (Figure 2A). In the case of flooding, with menisci present at the mouths of all regional alveoli, or in common ducts, the region as a whole should be diminished below its normal, aerated size. Septa within the region should be shorter than when the regions is aerated and normal-sized and, with equal *P*_*LIQ*_ to each side, subject to Δ*P*_*S*_ = 0 cm H_2_O and planar. The septa should be subject to reduced stress. Septa composing the perimeter of the flooded region will be intervening septa. If the region as a whole is not too diminished in size, the septa may be sites of stress concentration. (Significant regional diminishment may move the septal endpoints close enough that even bowing does not cause over-distension). And radiating septa splaying outward from the injured region should be sites of stress concentration. Computational modeling indicates that radiating septal stress concentrations are greater and penetrate further into surrounding aerated parenchyma for a multi-alveolar injured region than for a single injured alveolus (Albert et al., 2019). Mead et al.’s (1970) classic model suggests that *P*_*D*_ around a multi-alveolar injured region exceeds the recoil pressure of the lungs. Further, two injured regions in relatively close proximity would be expected to amplify stress concentrations in radiating septa bridging through healthy tissue between the regions. How injured-region size and distance between injured regions affect stress in radiating septa between the regions has yet to be characterized.

For a region with heterogeneous flooding or collapse (Figure 2B), stress concentrations within the region should be higher than normal and higher than within a homogeneously flooded region. That is, there will be stressed intervening septa and stressed radiating septa (bridging between two flooded/collapsed alveoli) within a heterogeneously injured region. With the heterogeneously injured region containing injured/diminished and aerated/over-expanded alveoli, the region as a whole should be less diminished in size than if homogenously injured. Thus, intervening septa on the perimeter may well be a site of stress concentration. And radiating septa should be less stressed than those around a homogeneously injured region, though they may still be a site of stress concentration.

At the whole-lung scale, the dependent lung is often a gross region of extensive flooding or collapse (Gattinoni et al., 2001). (Both the flooded dependent lung and aerated non-dependent lung likely comprise, to greater or lesser degrees, sub-regions of homogeneously flooded or collapsed alveoli, heterogeneously flooded or collapsed alveoli, and aerated alveoli). Stress might be expected to be concentrated at the border between the dependent and non-dependent regions. However, CT data show the border region to exhibit an intermediate density between that of the dependent and non-dependent regions (Terragni et al., 2007). Thus, with the caveat that there could be a few layers of stressed, over-distended alveoli or septa in the border region that are not detectable by CT, there is no evidence for stress concentration at the border. There is, however, evidence that diminishment of the flooded dependent lung is marked enough to cause ventilation-induced over-distension injury throughout the non-dependent lung. In a saline lavage-induced high *T* injury model in rats, high tidal volume, *V*_*T*_/low positive end-expiratory pressure (PEEP) ventilation caused atelectasis in the dependent lung and greater alveolar injury in the non-dependent than in the dependent lung (Tsuchida et al., 2006). The larger the injured area, it appears, the further acting the effect of interdependence.

### Alveolar Compliance and Septal Stress

Consideration of alveolar compliance yields additional insight into the stresses applied to intervening and radiating septa. The injured lung exhibits reduced compliance. Contrary to expectation, however, flooding alone does not alter alveolar compliance. When a single alveolus in a healthy lung is flooded and *T* remains normal, the alveolus becomes diminished in size but maintains normal compliance (Figure 1D) (Perlman et al., 2011). Adjacent aerated alveoli, due to interdependence, become enlarged and, due to the non-linear stress-strain relation of septal tissue (Perlman and Wu, 2014), exhibit reduced compliance.

**FIGURE 3:**
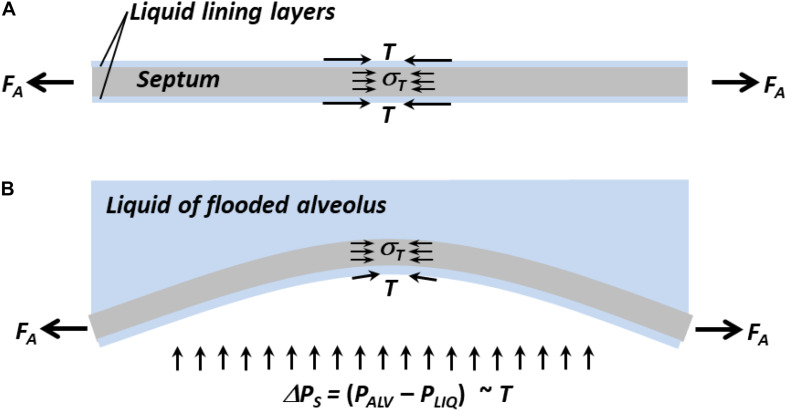
Forces acting on septa. **(A)** Normal planar septum between two aerated alveoli. Distending force *F*_*A*_ applies axial tension. Interfacial surface tension, *T*, and tissue stress, *σ_*T*_*, counter *F*_*A*_. **(B)** Intervening septum separating flooded (top) from aerated (bottom) alveolus. Due to *ΔP_S_*, which is ∼*T*, septum bows into flooded alveolus. Inflation-induced increases in *F*_*A*_ and *σ_*T*_* tend to reduce bowing. Inflation-induced increase in *T* acting along septum tends to reduce bowing, but inflation-induced increase in *T* also increases *ΔP_S_*, which tends to increase bowing. Net result is that inflation sometimes increases bowing (Figure 1A).

Lung inflation increases *F*_*A*_ and extends septa. In planar septa, the increase in *F*_*A*_ is balanced by increases in *T* and tissue stress, *σ_*T*_* (Figure 3A). In intervening septa (Figure 3B), the increase in *F*_*A*_ tends to pull apart the septal endpoints and reduce bowing, and the increase in *σ_*T*_*, which still counters *F*_*A*_, likewise tends to reduce bowing. However, the inflation-induced increase in *T* has two opposing effects. Acting along the septum, like σ*_*T*_*, elevated *T* tends to reduce bowing. Elevated *T* also increases Δ*P*_*S*_, which tends to increase bowing. Thus, lung inflation sometimes increases bowing of intervening septa (Figure 1A).

Inflation-induced increase in intervening septal bowing into a flooded alveolus/out of an adjacent aerated alveolus should tend to decrease compliance of the flooded alveolus and increase compliance of the adjacent aerated alveolus. The perimeter of a single flooded alveolus is formed entirely by intervening septa. For a single flooded alveolus to exhibit the same compliance as in its prior aerated state (Figure 1D, left) requires that inflation, at the same time as increasing inward bowing of some intervening septa, cause the septal junctions at the alveolar corners to move outward. If inflation moves apart the endpoints of an intervening septum while simultaneously increasing bowing of the septum, then inflation increases stress in the septum. The perimeter of an adjacent aerated alveolus includes one intervening septum and two radiating septa. For an adjacent aerated alveolus to exhibit reduced compliance compared with its state prior to flooding of its neighbor (Figure 1D, right) requires that inflation, at the same time as increasing outward bowing of the intervening septum, cause less distension of the radiating septa than prior to flooding of the neighbor. That is, the radiating septa must be stiffer than prior to flooding of the neighbor. Thus, the observation of flooding-induced decrease in compliance in adjacent aerated alveoli supports the prediction from modeling (Mead et al., 1970; Albert et al., 2019) that stress is concentrated in radiating septa.

### Dynamic, *T*-Dependent Stress Concentrations

Oftentimes, there is a threshold opening pressure, *P*_*O*_, required at the airway entrance to open flooded, collapsed, or injured lung regions, and *P*_*O*_ ∼ *T*. For air to enter and cause initial inflation of fluid-filled fetal lungs requires a *P*_*O*_ that is proportional to *T* (Enhörning and Robertson, 1972). Similarly, for air to enter a fluid-filled region of an injured lung or peel apart apposed septa separated by a thin liquid layer in a collapsed alveolus should require a *P*_*O*_ that is proportional to *T* (Gaver et al., 1990; Rühl et al., 2019). When PEEP < *P*_*O*_ < PIP, intervening and radiating septa around a flooded or collapsed region will be subject to maximal stress, in each ventilation cycle, just before the airway entrance pressure, *P*_*AW*_, exceeds *P*_*O*_. Thus, maximal stress in the septa surrounding an injured region that cyclically reopens should be proportional to *T*.

Interestingly, increasing *P*_*AW*_ above *P*_*O*_ could be a moving goal post. Once inflation commences, newly opened interfacial area may increase *T* and thus the *P*_*O*_ required for continued inflation. This scenario is one of negative feedback that may underlie incomplete opening of flooded or injured lung regions, particularly in cases of high *T* (Enhörning and Robertson, 1972).

### Experimental Evidence That Heterogeneous Flooding Causes Injurious Stress Concentrations

To determine whether stress concentrations in regions of heterogeneous flooding are injurious, my group performed experiments in isolated rat lungs.

**FIGURE 4:**
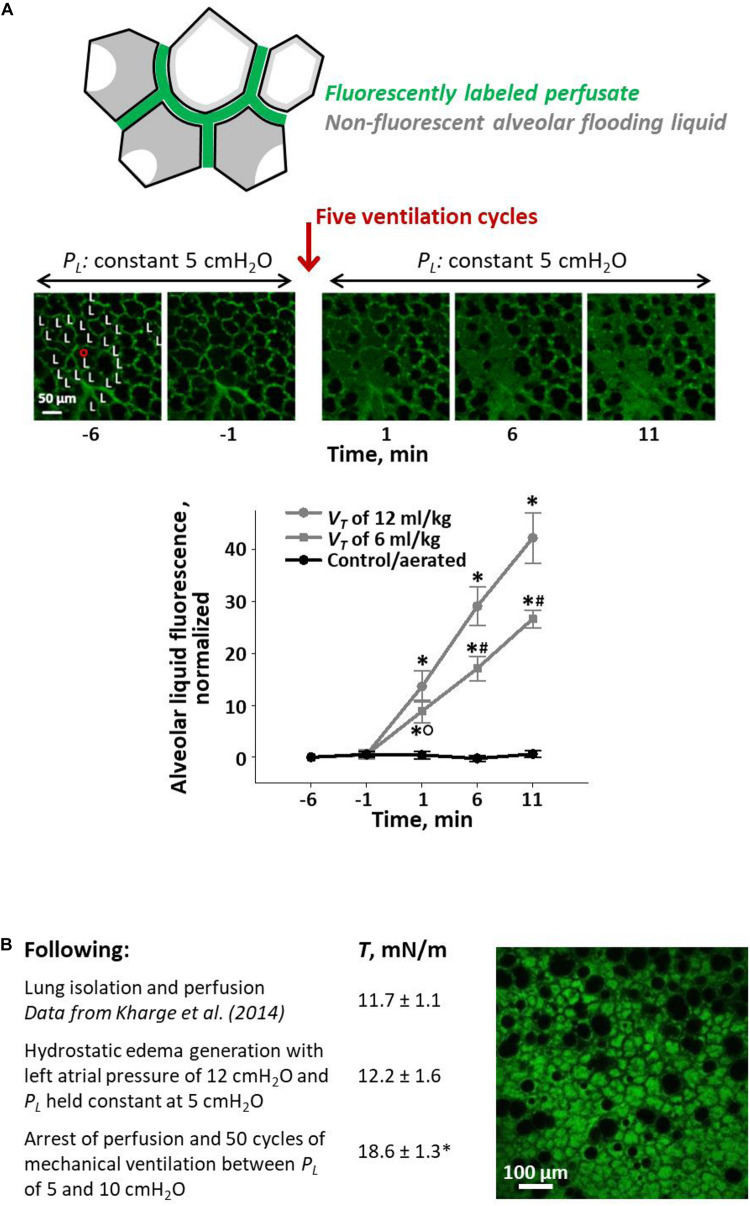
Ventilation-induced lung injury (VILI) assays. **(A)** Local VILI assay of ventilation-induced injury to alveolar–capillary barrier in the presence of heterogeneous alveolar flooding. Top schematic: Isolated lung is perfused with fluorescein-labeled blood/5% albumin mixture. A glass micropipette is used to puncture a surface alveolus and infuse non-fluorescent 3% albumin solution, which causes heterogeneous alveolar flooding in a local region. Menisci are present in flooded alveoli. Middle images: With *P*_*L*_ held at 5 cm H_2_O, the region is imaged by confocal microscopy over 5-min baseline period. “L” labels alveoli flooded with non-fluorescent liquid. Then, five ventilation cycles are administered with an end-expiratory *P*_*L*_ equivalent to an *in vivo* positive end-expiratory pressure (PEEP) of 10 cm H_2_O and a tidal volume, *V*_*T*_, of 12 ml/kg. Following ventilation, *P*_*L*_ is returned to 5 cm H_2_O, and the region is imaged for 10 additional minutes. Bottom graph: plot of increase above baseline of normalized fluorescence in alveolar liquid vs. time. Gray: experimental, heterogeneously flooded regions in lungs subjected to five ventilation breaths with 10 cm H_2_O PEEP and *V*_*T*_ as specified (fluorescein intensity quantified in alveolar flooding liquid). Black: control, aerated regions in lungs subjected to five ventilation breaths with 10 cm H_2_O PEEP and 6 or 12 ml/kg *V*_*T*_ (data for two *V*_*T*_ groups combined; fluorescein intensity quantified in liquid lining layer). Statistics: ^∗^*p* < 0.01 vs. baseline; #*p* < 0.01 vs. both other groups at the same time point; °*p* < 0.01 vs. control group at the same time point. Figure modified, with permission, from Wu et al. (2014). **(B)** Global VILI assay of ventilation-induced increase in *T* in the presence of heterogeneous alveolar flooding. Table: summary of experimental protocol and *T-*values, all determined at *P*_*L*_ of 15 cm H_2_O, at different time points. Statistics: ^∗^*p* < 0.05 vs. *T*-values at two earlier time points. Image: representative view of alveolar flooding pattern on surface of lungs at final time point after induction of VILI and elevation of *T*. Alveolar edema liquid labeled by fluorescein administered to the perfusate. Figure modified, with permission, from Wu et al. (2017).

We developed a local VILI assay in heterogeneously flooded regions of healthy lungs, in which alveolar-capillary barrier permeability was initially normal, and used increase in permeability as an indicator of injurious stress level (Figure 4A) (Wu et al., 2014). In isolated perfused rat lungs, we labeled the perfusate with fluorescein. By micropuncture of a surface alveolus, we infused a non-fluorescent solution of 3% albumin in normal saline into a local region of the parenchymal airspace. The infusion resulted in heterogeneous alveolar flooding. In control regions, we omitted the micropuncture and infusion such that all alveoli remained aerated. Following this preparation, we held the lungs at a constant transpulmonary pressure, *P*_*L*_, of 5 cm H_2_O and imaged a flooded or control region at two baseline time points; provided five ventilation breaths with a PEEP of 0–20 cm H_2_O (airway pressure adjusted to account for isolated lung with pleural pressure of zero) and a *V*_*T*_ of 6 or 12 ml/kg; and returned the lungs to a constant *P*_*L*_ of 5 cm H_2_O for 10 min of post-ventilation imaging. In control regions without alveolar flooding, thus without septal stress concentrations, fluorescein concentration in the alveolar liquid lining layer remained constant at the baseline level, regardless of ventilation settings, over the 10-min post-ventilation period. In flooded regions with stress concentrations, just five breaths with the gentlest ventilation settings caused fluorescein to leak into the alveolar flooding liquid. Further, fluorescein concentration increased continuously over the 10-min post-ventilation period, indicating that transient ventilation caused a sustained increase in barrier permeability. Increasing PEEP or *V*_*T*_ increased the rate of fluorescein entrance into the alveolar liquid.

In a different, global VILI assay, we assessed ventilation-induced increase in *T* in the presence of heterogeneous flooding throughout the lungs (Figure 4B) (Wu et al., 2017). Our protocol was as follows. First, in isolated and perfused but unventilated rat lungs, we raised left atrial pressure to generate hydrostatic edema. We obtained heterogeneous alveolar flooding. Second, we arrested perfusion and determined *T* at the meniscus of a flooded surface alveolus according to our established method (Kharge et al., 2014). Briefly, following two ventilation cycles between *P*_*L*_ of 5 and 15 cm H_2_O, we held the lungs at constant *P*_*L*_ of 15 cm H_2_O, determined *P*_*ALV*_ with a tracheal transducer, determined *P*_*LIQ*_ by servo-nulling pressure measurement, determined three-dimensional interfacial radius of curvature by confocal imaging, and calculated *T* according to the Laplace relation. We found that hydrostatic flooding did not alter *T* from normal. [We determined normal *T* in healthy lungs by applying the same method to the curved interface of the liquid lining layer in the corners of aerated alveoli (Kharge et al., 2014).] Finally, with perfusion still arrested, we gently ventilated the heterogeneously flooded lungs 50 times between *P*_*L*_ of 5 and 10 cm H_2_O and then again determined *T*. The ventilation increased *T* by 52%; we speculate that, as discussed below, debris from damaged cells caused the increase in *T*. Thus, again, ventilation exacerbated stress concentrations present due to heterogeneous alveolar flooding and led to lung injury.

We have not, to date, been able to distinguish between injury to intervening vs. radiating septa. However, our studies demonstrate that stress concentration levels are injurious in at least one of the two types of septa.

### Spatial Propagation of Injury

Computed tomography analysis, in an acid-induced ARDS animal model and clinical ARDS, showed that ventilation causes injured areas to grow larger over time (Cereda et al., 2017). That over-stressed intervening and radiating septa are located at the perimeters of injured regions suggests that injured areas might grow larger through over-expansion of initially normal alveoli at the borders of injured regions. In this first scenario, high maximum *T* might be especially problematic. Alternatively, ventilation-induced exacerbation of the condition in an injured region—for example, by transiently high-pressure gradients imposed when *P*_*AW*_ > *P*_*O*_ re-expands an injured region (Bilek et al., 2003)—might exacerbate already-elevated barrier permeability and lead to additional extravasation of liquid. The new liquid might extend, and thus generate stress concentrations, beyond the originally-injured area, thus leading to outward expansion of the injured area. In this second scenario, as *P*_*O*_ ∼ *T*, high minimum *T* might be especially problematic. The mechanism of spatial injury propagation remains to be determined.

## Surface Tension in ARDS

Bronchoalveolar lavage fluid (BALF) from ARDS patients exhibits increased adsorption time and elevated minimum surface tension, *T*_*MIN*_, when tested *in vitro* in Langmuir–Wilhelmy troughs and pulsating bubble surfactometers (PBSs) (Hallman et al., 1982; Gregory et al., 1991). These results have overwhelmingly been attributed to the presence of plasma proteins in edema liquid. My group (Kharge et al., 2014) and Holm et al. (1988, 1999) have suggested, as detailed below, that the results are attributable co-adsorption of surfactant and plasma proteins in *in vitro* tests and shown that when an intact surfactant monolayer is present before plasma proteins enter the subphase, as occurs in the lungs, plasma proteins do not alter *T*. Other causes of elevated *T* in ARDS have been proposed, and indeed, Günther et al. (1996) showed that ARDS BALF from which proteins were removed still exhibited elevated *T*. However, other purported *T-*raising substances have also generally been tested under co-adsorption conditions. And even if a monolayer is formed first, another consideration is that, in the absence of transformation by rapid compression, *in vitro* monolayers lack the metastability exhibited by surfactant in the lungs (Horie and Hildebrandt, 1971; Smith et al., 2004). The ability of other substances to raise *T* of an intact and metastable monolayer requires testing.

### Plasma Components

Proteins are the plasma component in alveolar edema liquid most widely cited as raising *T*. However, plasma proteins have only been shown to raise *T* by competitive adsorption and may not have the opportunity to adsorb in the lungs. With an initially clean interface in a trough with constant interfacial area or a PBS with constant bubble volume, plasma proteins that co-adsorb with surfactant raise *T* (Holm et al., 1988, 1999; Seeger et al., 1993). Proteins that co-adsorb with surfactant in a PBS also raise the *T*_*MIN*_ achieved by subsequent bubble pulsation (Seeger et al., 1993; Holm et al., 1999). With an initially-intact interfacial surfactant monolayer in a Langmuir–Wilhelmy trough, plasma proteins injected into the subphase raise *T*_*MIN*_ during subsequent 80% cyclic surface area compression (Holm et al., 1999; Warriner et al., 2002). In the Langmuir–Wilhelmy trough, plasma proteins should have the opportunity to co-adsorb after the monolayer is ruptured by compression to 20% of initial area or to adsorb at peak surface area when regions of clean interface are exposed by monolayer expansion into the two-dimensional gaseous phase (Warriner et al., 2002; Wüstneck et al., 2005). However, with an intact surfactant monolayer, plasma proteins lack access to the interface and fail to raise *T*. Holm et al. (1988) showed that albumin injection below a static, intact surfactant monolayer fails to raise *T*. Holm et al. (1999) also generated an intact monolayer in a PBS and then added albumin to the subphase by subphase exchange. Even with subsequent 50% cyclic surface area compression, albumin below the intact monolayer failed to raise *T*_*MIN*_. While 50% is just about the compression that begins to collapse the monolayer (Schürch et al., 1992; Holm et al., 1999), we estimate compression in the lungs to be, at most, ∼40% (Kharge et al., 2014). Further, the monolayer in the lungs is always in the liquid-condensed phase (Wüstneck et al., 2005; Kharge et al., 2014; Nguyen and Perlman, 2018) such that even maximal lung inflation should not expose clean interface. Accordingly, in isolated healthy rat lungs held above functional residual capacity (FRC), alveolar injection of 5% albumin solution or blood plasma does not raise *T*. That plasma fails to raise *T* in the lungs indicates that, as discussed previously (Kharge et al., 2014), no plasma protein is likely at physiologic concentration to raise alveolar *T* with an intact surfactant monolayer.

In injured lung regions, it is possible that the surfactant monolayer might collapse, and allow plasma protein adsorption, at the end of expiration. In rat lungs at FRC, *P*_*L*_ is in the range of 3.5–4.5 cm H_2_O (Schürch et al., 1976). With albumin present in alveolar flooding liquid, ventilating between *P*_*L*_ of 3 and 30 cm H_2_O failed to increase *T*; ventilating between *P*_*L*_ of 1 and 30 cm H_2_O caused a transient increase in *T* that was subsequently completely reversed by additional surfactant spreading (Kharge et al., 2014). In injured regions, local volume is certain to be below normal at FRC. Whether that volume is sufficiently low to enable plasma proteins to raise *T*, and whether any increase in *T* would be sustained, remains to be determined.

Another plasma component in alveolar edema liquid suspected of raising *T* is cholesterol. Native surfactant includes 5–10% cholesterol. Additional cholesterol has been mixed with surfactant by organic phase combination or, with cyclodextrin-facilitated solubilization, aqueous phase combination (Gunasekara et al., 2005; Lugones et al., 2018). Cholesterol-induced *T* elevation has been shown, following co-adsoprtion, to depend on concentration, requiring a nominal cholesterol fraction of ≥20%, and surfactant composition. However, cholesterol in blood plasma did not raise *T* of an intact surfactant monolayer in the lungs (Kharge et al., 2014).

### Debris From Damaged Cells

With elevated barrier permeability and occasional hemorrhagic regions in ARDS, cells including epithelial cells and RBCs are damaged, and their contents should be present in edema liquid (Henzler et al., 2011; Santos et al., 2012). Cellular components including hemoglobin (Hb) from RBCs and phospholipids—particularly unsaturated phospholipids and lysophospholipids—and cholesterol from cell membranes may interfere with surfactant function and raise *T* (Holm and Notter, 1987; Holm et al., 1991, 1999; Hite et al., 2005; van Meer et al., 2008). The ability of Hb and RBC membrane lipids to increase *T* depends on the quantities of these inhibitory factors relative to the quantity of surfactant present (Holm and Notter, 1987; Holm et al., 1991, 1999; Hite et al., 2005). In ARDS edema liquid, my group estimated phospholipid concentration to be 2 mg/ml ≈ 2.7 μmol/ml, based on the molecular weight of dipalmitoylphosphatidylcholine (Kharge et al., 2014). At a comparable 2 μmol/ml of lipids in whole lung surfactant, Holm and Notter (1987) found membrane lipids more inhibitory than Hb and >1 μmol/ml membrane lipids necessary to raise *T*_*MIN*_. Holm et al. (1999) also found lysophosphatidylcholine capable of raising *T* of an already-established intact surfactant monolayer. The high cholesterol content of cell membranes might also behave differently than plasma cholesterol and contribute to surfactant dysfunction (Gunasekara et al., 2005; van Meer et al., 2008; Lugones et al., 2018). Thus, cell debris may contribute to the elevated *T* of alveolar edema liquid.

### Surfactant Degradation

Surfactant lipids and proteins may be degraded by enzymes or reactive oxygen species (ROS). Inflammatory cells release secretory phospholipase A_2_ and certain bacteria, including *Pseudomonas*, release phospholipase C (Holm et al., 1991; Kitsiouli et al., 2009). Accordingly, in BALF from ARDS patients, elevated phospholipase levels and activity have been observed (Hallman et al., 1982; Seeds et al., 2012). Phospholipases could elevate *T* directly, by reducing the availability of *T-*lowering surfactant phospholipids. Additionally, the products of phospholipase activity, lysophospholipids and fatty acids, raise *T*_*MIN*_ in a PBS, perhaps by intercalating into and increasing fluidity of the monolayer (Holm et al., 1991, 1999; Hite et al., 2005). As mentioned, lysophosphatidylcholine, in particular, has been shown to raise *T* of an already-established intact surfactant monolayer (Holm et al., 1999). Thus, phospholipases in alveolar edema liquid may increase *T* in the lungs.

In ARDS, ROS from activated leukocytes and macrophages can attack the surfactant monolayer from below. Oxidation of phospholipids first transforms unsaturated hydrocarbon tails and then causes cleavage, resulting in lysophospholipids and fatty acids (Rodríguez-Capote et al., 2006). Oxidized phospholipids cause only a marginally greater increase in *T* than their unoxidized counterparts and, thus, might not increase *T* much more than any alteration in phospholipid composition due to contamination by lipids of degraded cell membranes. However, ROS-induced generation of lysophospholipids, which increase *T* of an intact surfactant monolayer, has the potential to raise *T*. Oxidation also alters the form and function of surfactant proteins B and C (SP-B and -C) (Rodríguez-Capote et al., 2006). For example, oxidation causes depalmitoylation of SP-C, which is known to inactivate SP-C (Gustafsson et al., 2000). Nonetheless, it is almost entirely oxidation of SP-B that results in elevated *T* (Rodríguez-Capote et al., 2006). The effect of ROS on surfactant proteins has been tested with co-adsorption of reconstituted surfactant components including already-oxidized proteins but not with ROS application below an intact surfactant monolayer.

Surfactant protein alteration might also contribute to the acceleration, in ARDS, of normal surfactant recycling. Surfactant isolated from BALF by centrifugation partitions into surface-active large aggregates and surface-inactive small aggregates. In ARDS, the ratio of large-to-small aggregates decreases (Günther et al., 2002). In addition, conversion of large-to-small aggregates has been shown to be facilitated by serine-active carboxyl esterases (Ruppert et al., 2003). However, whether one of the surfactant proteins might be a substrate of the esterases is not known.

### Gastric Liquid Aspiration

Aspiration of acidic gastric contents is a common cause of ARDS. In this situation, low pH gastric contents pass through the airways and mix with airway liquid, *en route* to the alveoli.

Low pH does not appear directly to alter surfactant function. In a PBS, pH as low as 2 did not alter *T*_*MIN*_ of the hydrophobic fraction of calf lung surfactant (Haddad et al., 1994). In addition, when we administered pH 1.9 HCl solution by direct micropuncture to surface alveoli of isolated rat lungs, the solution raised alveolar *T* over a period of ∼30 min (Nguyen and Perlman, 2018). The time delay in the lungs suggests that low pH might raise *T* indirectly, perhaps by damaging the alveolar epithelium and releasing cell components that subsequently interfere with surfactant function.

Administration of pH 1.9 hydrochloric acid (HCl) solution via the trachea also raised alveolar *T* in isolated rat lungs. Surprisingly, however, this effect was not pH dependent. Tracheal instillation of pH 5.0 normal saline caused the same increase in alveolar *T* (Nguyen and Perlman, 2018). The *T*-raising effect of HCl or saline instillation was not due to surfactant dilution as direct alveolar injection of saline, which should cause greater dilution, did not alter *T* from normal. Further, even tracheal instillation of the surfactant Infasurf raised alveolar *T*. Thus, we suspected the involvement of airway mucins and, indeed, injection into surface alveoli of tracheal lavage liquid raised alveolar *T*. Addition to the tracheal lavage liquid of HCl or calcium, both of which aggregate mucins, reduced or eliminated the effect of tracheal lavage liquid on alveolar *T*. And, following tracheal saline administration, alveolar liquid sampled directly by vacuum suction through a glass micropipette contained mucin 5B. Thus, we demonstrated that tracheal liquid instillation raises alveolar *T* by washing *T-*raising airway mucins to the alveolus.

In gastric aspiration, the effects of pH and mucins should tend to balance one another. If gastric contents mix significantly with airway liquid, then they should be partially buffered but also collect mucins (Kim et al., 2014; Nguyen and Perlman, 2018). The higher pH might not be injurious, but the mucins might elevate *T* (Nguyen and Perlman, 2018). Alternatively, if there is relatively little mixing with airway liquid, there will be relatively little buffering, and pH will remain low. The low pH will tend to aggregate mucins such that the mucins might not contribute to elevated *T* (Hong et al., 2005; Nguyen and Perlman, 2018). The low pH, itself, should lead to elevated *T*, albeit perhaps indirectly via debris from damaged epithelial or other cells. Regardless of the degree of mixing between gastric contents and airway liquid, gastric aspiration is likely to raise alveolar *T*.

## Means of Reducing *T*-Dependent VILI

Surface tension is believed to be elevated in ARDS and is further increased by lung inflation during ventilation. And the static and dynamic stress concentrations described above, which likely contribute to VILI, are proportional to *T*. Thus, lowering *T* should reduce the VILI.

### Means of Reducing *T*

Surfactant therapy has been tested for ARDS but, in six randomized controlled clinical trials, failed to reduce mortality (Brower and Fessler, 2011). Certainly, surfactant could lower *T* and yet fail to reduce mortality. However, surfactant therapy only improved oxygenation significantly in one of the six trials and marginally in one other and did not improve compliance in either of the two trials in which compliance was assessed (Weg et al., 1994; Anzueto et al., 1996; Gregory et al., 1997; Spragg et al., 2004, 2011; Kesecioglu et al., 2009), suggesting that it is a challenge for exogenous surfactant to lower alveolar *T*. Problematic surfactant dosage, administration technique, and formulation have been discussed as possible causes of the failure of surfactant therapy in ARDS (Spragg et al., 2011; Kazemi et al., 2019). My group has identified two new potential causes for the failure of surfactant therapy. First, surfactant delivery appears to be problematic. As detailed above, tracheal instillation of any liquid washes *T-*raising mucins to the alveolus. Thus, tracheal instillation of surfactant, contrary to expectation, *raises* alveolar *T* (Nguyen and Perlman, 2018). [Lower mucin production and more airspace liquid to dilute mucins may enable surfactant therapy efficacy in NRDS (Buisine et al., 1999)]. Second, when *T* is elevated in ARDS, adding more surfactant may fail to reduce *T*. In isolated rat lungs, we modeled acid-aspiration ARDS by administering pH 1.9 HCl solution to the alveolus by direct micropuncture or indirect tracheal instillation (Nguyen and Perlman, 2018). Both administration methods raised alveolar *T*. In both cases, however, subsequent alveolar delivery of mucin-free Infasurf by micropuncture failed to reduce *T*. Whether surfactant, if it could to be delivered mucin-free in a clinical setting, would be effective in other forms of ARDS remains to be determined.

With our *T*-determination method, we identified a potential alternative means of reducing *T* in ARDS. We found that that the non-toxic dye sulforhodamine B (SRB) acts in conjunction with albumin present in edema liquid to improve the efficacy of native lung surfactant and lower *T* 27% below normal in healthy lungs (Kharge et al., 2015). Thus, albumin, generally believed to be detrimental, might beneficially help lower *T*. Further, SRB, which has a molecular weight of 577 and is water soluble, might be administered intravenously. In this case, SRB would be expected to reach the alveolar liquid, along with albumin, specifically in injured regions where barrier permeability is elevated. Whether SRB will work in the presence of the various factors responsible for elevating *T* in ARDS remains to be determined.

### Effects of Reducing *T*

Reducing *T* should help reduce VILI via various mechanisms. Direct effects of lowering *T* are as follows. Lowering *T* should increase *P*_*LIQ*_ in flooded alveoli, thus reduce bowing of and stress concentrations in intervening septa. Lowering *T* should reduce the diminishment of flooded alveoli and may prevent or reverse alveolar collapse, thus should reduce stress concentrations in radiating septa. And reducing stress concentrations in intervening or radiating septa should reduce VILI. Indeed, with our local VILI assay (Figure 4A), we demonstrated that lowering *T*, including by administration of SRB, reduced ventilation-induced barrier injury (Wu et al., 2014; Kharge et al., 2015).

Lowering *T* should also indirectly reduce stress concentrations in radiating septa. That is, lowering *T* should reduce the *P*_*O*_ required for air to enter a flooded region or to peel open the apposed septa of a collapsed region (Enhörning and Robertson, 1972; Gaver et al., 1990). Thus lowering *T*, by lowering *P*_*O*_, should reduce the peak cyclic stress applied, just before reopening, to radiating septa around the injured region.

Further, lowering *T* may reduce VILI by promoting re-aeration of previously flooded or collapsed alveoli. Low *T*-induced re-aeration of a flooded alveolus is a rapid process in which liquid that has been sequestered in the alveolus redistributes, more equitably, across regional alveoli. In regions of heterogeneous alveolar flooding or collapse (Figures 2B, 4), re-aeration should reduce the degree of heterogeneity, thus, the number of intra-regional stress concentrations. For example, in regions of heterogeneous alveolar flooding, considering that bowed intervening septa are likely more stressed than planar septa separating two aerated or two flooded alveoli, we quantified heterogeneity as the percentage of all septa that were intervening septa (Wu et al., 2017). (In this basic analysis, we did not account for the presence of stressed radiating septa within injured regions). We found that heterogeneity peaked when ∼50% of alveoli were flooded. Thus with heterogeneous flooding, reducing the number of flooded alveoli should reduce the number of intervening septa in which stress is concentrated and, in turn, VILI. (If >50% of alveoli are initially flooded, reducing the number of flooded alveoli should first exacerbate and then, once alveolar flooding decreases below 50%, relieve VILI). In regions with homogeneous alveolar flooding or collapse, re-aeration is most likely to occur on the periphery of the region (see below). As intervening and radiating septa around the perimeter of a homogeneously injured region are the sites of greatest stress concentration, re-aeration should, by reducing injured region perimeter length, reduce the number of septa in which stress is concentrated and reduce VILI.

Experimental evidence supports the possibility that lowering *T* may promote rapid re-aeration that could be beneficial in the time period before re-absorption causes the permanent re-aeration of ARDS resolution. Though the mechanism of rapid re-aeration has yet to be fully elucidated, my group has made observations that inform an understanding of the process (Kharge et al., 2015; Wu et al., 2017). (i) Flooded alveoli generally remain flooded and, at normal *T*, lung inflation does not generally cause re-aeration. However, occasionally, a flooded alveolus will spontaneously re-aerate. (ii) When *T* is reduced by SRB administration and the lungs are gently ventilated, the frequency of re-aeration increases. The direct effect of lowering *T* might be amplified by reduced *T* causing greater tissue stretch and thus contributing to enhanced surfactant secretion. (iii) It is also possible to promote re-aeration by accelerating deflation to increase momentum transfer to the flooding liquid and, essentially, catapult liquid out of alveoli. Accelerated deflation successfully reduces the number of flooded alveoli, and thus flooding heterogeneity, only when *T* is normal (below-normal *T* should be even better), and PIP is moderate, such that *T* at peak inflation is not too high. (iv) When *T* is elevated ∼50% above normal in our model of Figure 4B, accelerated deflation still propels liquid out of flooded alveoli, but the high *T* keeps the liquid aggregated. The liquid transfers *en mass* to a neighboring alveolus that was previously aerated and the number of flooded alveoli remains unchanged. (Consistent with the hydrostatic origin of flooding in this model, the observation of liquid transfer between alveoli indicates that alveoli are truly flooded, not collapsed. That is, flooding liquid volume is greater than the aggregated liquid lining layer volume that would be found in the center of a collapsed alveolus). (v) Finally, whether spontaneous or prompted by reduced *T* or accelerated deflation, we do not observe re-aeration of flooded alveoli surrounded entirely by other flooded alveoli but, rather, always of flooded alveoli that were in contact with one or more aerated alveoli. We interpret that flooded alveoli that are adjacent to at least one aerated alveolus are in a state of *T-*dependent unstable equilibrium.

**FIGURE 5:**
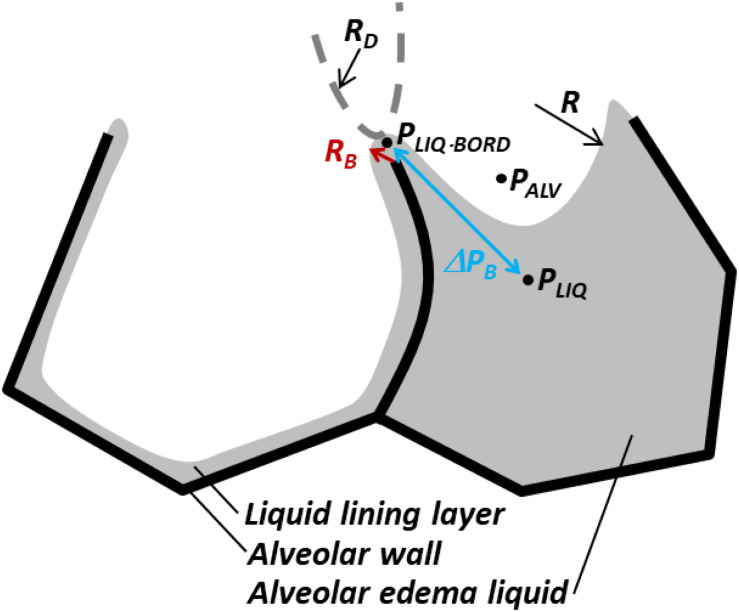
Pressure barrier, Δ*P*_*B*_, traps liquid in flooded alveoli. According to the Laplace relation, pressure in liquid below meniscus is *P*_*LIQ*_ = *P*_*ALV*_ *– 2*T*/R* < *P*_*ALV*_, where *R* is the meniscus radius. At the border between two adjacent alveoli, shared septum terminates at alveolar duct or sac, and liquid phases of alveoli are presumably continuous. Thus, in the plane of the figure, the interface has a small convex radius *R*_*B*_ at the border of the flooded alveolus. In the plane perpendicular to the figure, the end of the septum and the interface should have larger concave radius *R*_*D*_ of duct or sac, indicated by dashed gray curve. Again, according to the Laplace relation, the liquid phase pressure at the border of the alveolus is *P_LIQ_*._*BORD*_ = *P*_*ALV*_ + *T*(1/*R*_*B*_ – 1/*R*_*D*_) > *P*_*ALV*_. Thus, pressure barrier Δ*P*_*B*_ = *P_LIQ_*._*BORD*_ – *P*_*LIQ*_ = *T*(1/*R*_*B*_ – 1/*R*_*D*_ + 2/*R*) > 0 traps liquid in the flooded alveolus. As Δ*P*_*B*_ ∼ *T*, lowering *T* lowers Δ*P*_*B*_ and increases the likelihood of liquid escape from the flooded alveolus, i.e., the likelihood of re-aeration. Figure modified, with permission, from Kharge et al. (2015) and Wu et al. (2017).

The unstable equilibrium of flooded alveoli with aerated neighbors may be attributable to the antagonism across each intervening septum of outward-acting *P*_*D*_, applied by radiating septa (Figure 1C, right), vs. inward-acting Δ*P*_*S*_ (Figure 3B). We have not identified the circumstances in which outward *P*_*D*_ sometimes prevails over inward Δ*P*_*S*_, but note that a greater number of aerated neighbors make this occurrence more likely. Nevertheless, flooded alveoli typically remain flooded. This general stability is assisted by liquid pressure being greater at the border than in the center of flooded alveoli. That is, there is a pressure barrier, Δ*P*_*B*_, that opposes liquid flow out of flooded alveoli and that is proportional to *T* (Figure 5). By trapping liquid in flooded alveoli, Δ*P*_*B*_ tends to maintain the low *P*_*LIQ*_ in flooded alveoli and thus maintain the diminished size of flooded alveoli. However, lowering *T* reduces Δ*P*_*B*_ and increases the likelihood of re-aeration (Kharge et al., 2015). For this reason, accelerated deflation most effectively clears alveoli when PIP is moderate, such that high elastic recoil for momentum transfer is balanced with low peak *T* for maintaining a low Δ*P*_*B*_, and SRB administration increases the frequency of re-aeration during gentle ventilation (Kharge et al., 2015; Wu et al., 2017).

The existence of a pressure barrier is also supported by—and helps to explain—the difference in efficacy between two alternative recruitment maneuvers for initial inflation of immature fetal lungs. In a preterm lamb model, Tingay et al. (2019) showed that a gradual recruitment maneuver increases lung compliance more than an abrupt maneuver. This result might be explained by greater alveolar aeration at moderate *P*_*L*_ values—due to a moderate *T* and consequently moderate Δ*P*_*B*_—in the course of the gradual maneuver. Although Tingay et al.’s (2019) direct comparison appears to be unique, other studies suggest that gradual recruitment may also result in greater re-aeration in ARDS. First, ARDS lung volume increased more in response to inflation from *P*_*L*_ of 19 to 28 cm H_2_O than from 28 to 40 cm H_2_O (Cressoni et al., 2017). Second, in an ARDS recruitment maneuver comprising a 7-s ramp up to a sustained 40 cm H_2_O, essentially all of the effected volume increase occurred within the first 10 s after the ramp up period (Arnal et al., 2011). Finally, the response, in ARDS patients, to an abrupt 40 cm H_2_O recruitment maneuver was inversely proportional to extravascular lung water index (Smetkin et al., 2012)—suggesting that high pressure may not effectively clear fluid from edematous alveoli, perhaps due to a high Δ*P*_*B*_. A gradual recruitment maneuver might be more effective than an abrupt maneuver.

Re-aeration, by distributing liquid equitably between alveoli and thereby reducing the prevalence of stress concentrations, should reduce VILI. The balance between entering air (promoted by a high *P*_*AW*_ > *P*_*O*_) and escaping liquid (promoted by a low *P*_*L*_ that maintains a low *T* and low Δ*P*_*B*_) that causes re-aeration of a flooded alveolus remains to be determined. Such an understanding of the mechanism of flooded alveolar re-aeration might lead to new ventilation and recruitment maneuver protocols for maintaining an open lung.

## Discussion and Conclusion

The above analysis stems from observations of *T* effects on flooded alveolar mechanics and speculates about consequences not yet investigated experimentally. The speculation, however, has limitations. First, the experimental observations were made in alveoli that were generally flooded with proteinaceous model edema liquid but that were otherwise, at least prior to ventilation, normal. The influence of pre-existing injury on flooded alveolar mechanics is not known. For example, whether flooded injured alveoli maintain normal compliance is not known. Second, discussion of *T* effects in regions of alveolar collapse is speculative. Alveolar collapse in a lung injury model has not, to date, been identified by microscopy on the surface of isolated lungs. Thus, collapse has not been studied, like flooding, with high resolution and tracking across varied inflation pressures. Third, the mechanism of flooded alveolar re-aeration, which should reduce the prevalence of stress concentrations, remains to be elucidated. And the feasibility of permanently re-aerating injured alveoli in ARDS is not known. Nonetheless, evidence suggests that lowering *T* should reduce alveolar septal stress concentrations and that lowering *T* in conjunction with application of gradual recruitment maneuvers may improve lung re-aeration. And both interventions should reduce VILI. Treatment with SRB appears a promising avenue of investigation for lowering *T* and VILI in ARDS.

## Ethics Statement

Ethical review and approval was not required for the animal experiments because this manuscript presents animal data from prior studies, for which IACUC approval was previously obtained, but not any new animal data.

## Author Contributions

CP conceived of the subject matter for and wrote this manuscript.

## Conflict of Interest

The authors declare that the research was conducted in the absence of any commercial or financial relationships that could be construed as a potential conflict of interest.
